# Criteria to Qualify Microorganisms as “Probiotic” in Foods and Dietary Supplements

**DOI:** 10.3389/fmicb.2020.01662

**Published:** 2020-07-24

**Authors:** Sylvie Binda, Colin Hill, Eric Johansen, David Obis, Bruno Pot, Mary Ellen Sanders, Annie Tremblay, Arthur C. Ouwehand

**Affiliations:** ^1^Danone Nutricia Research, Palaiseau Cedex, France; ^2^APC Microbiome Ireland, University College Cork, Cork, Ireland; ^3^Emerging Technologies, Chr. Hansen A/S, Hørsholm, Denmark; ^4^Danone Nutricia Research, Palaiseau Cedex, France; ^5^Science Europe, Yakult Europe BV, Almere, Netherlands; ^6^International Scientific Association for Probiotics and Prebiotics, Centennial, CO, United States; ^7^Rosell Institute for Microbiome and Probiotics, Montreal, QC, Canada; ^8^Global Health and Nutrition Sciences, DuPont Nutrition and Biosciences, Kantvik, Finland

**Keywords:** probiotic definition, criterion, live microbes, *Lactobacillus*, *Bifidobacterium*, identification, safety, health efficacy

## Abstract

Still relevant after 19 years, the FAO/WHO definition of probiotics can be translated into four simple and pragmatic criteria allowing one to conclude if specific strains of microorganisms qualify as a probiotic for use in foods and dietary supplements. Probiotic strains must be (i) sufficiently characterized; (ii) safe for the intended use; (iii) supported by at least one positive human clinical trial conducted according to generally accepted scientific standards or as per recommendations and provisions of local/national authorities when applicable; and (iv) alive in the product at an efficacious dose throughout shelf life. We provide clarity and detail how each of these four criteria can be assessed. The wide adoption of these criteria is necessary to ensure the proper use of the word probiotic in scientific publications, on product labels, and in communications with regulators and the general public.

## Introduction

Consumers are increasingly interested in maintaining health through food and dietary supplements. Use of evidence-based approaches to improve diets and lifestyles is a trend that continues to grow. This has generated an ever more varied market of foods and supplements, especially those containing probiotics. An expert consultation convened under the umbrella of the World Health Organization (WHO) and the Food and Agriculture Organization proposed a useful definition of probiotics in 2001, which was later refined in 2014 for grammatical reasons to “live microorganisms that, when administered in adequate amounts, confer a health benefit on the host” (FAO/WHO, 2002; Hill et al., 2014). Hill et al. (2014) stipulated that probiotics must have “defined contents, appropriate viable count at end of shelf life and suitable evidence for health benefits,” and further stated that all probiotics must be “safe for their intended use.” These points were reiterated in 2018 by the International Scientific Association of Probiotics and Prebiotics (ISAPP) in a position statement (ISAPP, 2018). Even so, while the term “probiotic” is used widely in both food and supplement categories, it is often misused. Here we provide clarity to the minimum criteria needed for the proper use of the term probiotic. This is especially useful at a time when new “biotic” names are being introduced into the global vernacular (e.g., terms such as pharmabiotic, psychobiotic, postbiotic, synbiotic, and others).

**FIGURE 1:**
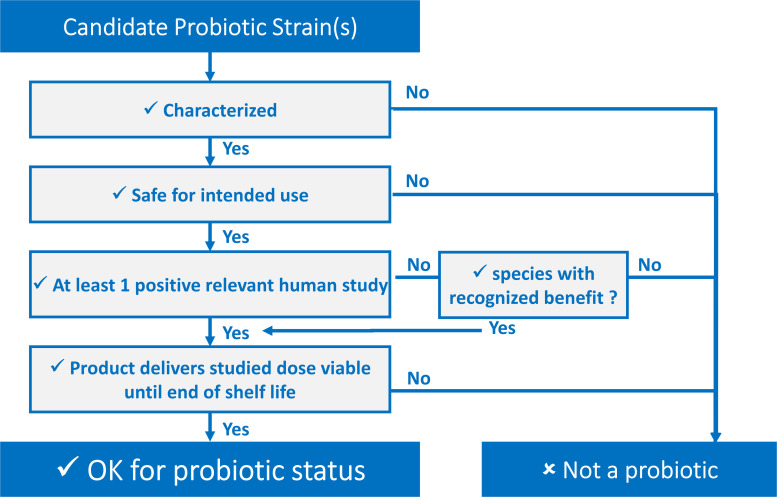
Decision tree to determine if a candidate probiotic fulfills the definition criteria.

Every part of the probiotic definition is important and can be “transposed” into easy-to-use criteria. Defining these criteria has been a key objective of different stakeholders in the probiotic field. These criteria can be presented as a decision tree, shown in Figure 1, serving as a tool for determining whether or not a candidate strain, or combination of strains, qualifies for probiotic status regardless of the final application. Further, they can be presented in a checklist fashion, such as in this ISAPP infographic (ISAPP, 2019) or as a list of “commandments” as has been suggested by Toscano et al. (2017).

The definition of probiotic contained herein is not restricted to traditional probiotics. Certainly, innovation will lead to candidate probiotics being isolated from novel sources, with currently unanticipated functions, and exciting, new health benefits. These so-called next generation probiotics, which may be conceptualized in some cases as live biotherapeutics, are not precluded under this definition. However, depending on the intended use, appropriate safety, legal and ethical matters must be addressed in the development of such probiotics, such as complying with the Nagoya protocol (Johansen, 2017) where applicable and in the case of isolating microbes from humans ensuring appropriate informed consent.

Notwithstanding earlier publications, there is a need to clearly and meticulously stipulate these criteria and provide details about achieving them without going into the specifics of potential mechanistic requirements. In short, probiotic strains must be (i) sufficiently characterized; (ii) safe for the intended use; (iii) supported by at least one positive human clinical trial conducted according to generally accepted scientific standards; and (iv) alive in sufficient numbers in the product at an efficacious dose throughout shelf life.

## Probiotic Is Sufficiently Characterized

The key component of correct probiotic characterization is proper strain identification and naming. Probiotic strains should be named according to the currently valid bacterial nomenclature, based on the International Code of Nomenclature (Parker et al., 2019). An updated list of prokaryotic names with standing in nomenclature is available at http://www.bacterio.net/ (Parte, 2018).

Identification of probiotic microbes entails determining that a strain belongs to an established, named genus and species, and subspecies for species in which subspecies have been described. Since some probiotic activities might be strain specific, a proper typing of the strain is furthermore required. Proper strain designation is therefore composed of two main parts: the official genus, species (and subspecies) names, according to the nomenclatural rules, followed by a strain designation which could be the catalog number of a recognized culture collection or a commercial strain designation. For this purpose, we recommend that the strain should be deposited in a recognized culture collection, for safe-keeping and so that the strain is available for research purposes, but not necessarily for commercial use. The use of multiple strain designations for a single strain should be avoided as this is a cause of confusion. Manufacturers should also ensure maintenance of genetic purity of their strains so that products contain the same strain with the same properties over time.

Identification technologies may vary according to the organism and will develop over time, but several molecular methods are currently widely applicable as phenotypic techniques alone are insufficient for proper identification. Sequencing of the 16S ribosomal DNA is a well-known and reliable way to identify species, assuming reliable reference sequences are used. The sequence obtained can first be matched to large reference databases that cover almost the entire known bacterial diversity, but final validation is preferably done using curated databases such as PATRIC (Wattam et al., 2017). In the case of doubt or for a more detailed identification, involving other molecular and phenotypic traits, Bergey’s Manual of Determinative Bacteriology can be consulted.^1^ Further, Mattarelli et al. (2014) described the “Recommended minimal standards for description of new taxa of the genera *Bifidobacterium*, *Lactobacillus* and related genera.” This is a useful resource for techniques that can be applied to proper identification as well.

The “gold standard” for strain identification is whole genome sequencing (WGS) including any extrachromosomal elements. A fully sequenced genome allows the identification of microbes to the species and strain level. Reference databases are available, e.g., at NCBI.^2^ Having a complete genome sequence has many advantages as it allows robust and precise strain specific identification and facilitates a search for the presence or absence of risk factors (see section “Probiotic is Safe for Intended Use” on safety). Furthermore, it will help to identify possible plasmids, common in some lactic acid bacteria and possibly important for probiotic activity. While WGS is the preferred method, other typing methods may allow comparison of individual strains. Multi-locus sequence typing or pulsed-field gel electrophoresis allow comparison of strains, but not *de novo* identification of species or genus. Identification should preferably be done by specialized or appropriately accredited laboratories, which can access the required reference databases and use current validated and calibrated methods and equipment. It should also be stressed that for probiotic mixtures, it is important that each individual strain in the mixture is properly identified, especially when each strain can be eligible for status as a probiotic.

The characterization of probiotic strains should support their probiotic activity. While clinical outcomes are required for a claim of probiotic functionality (see section “Probiotic is Supported by at Least one Human Clinical Trial According to Generally Accepted Scientific Standards” regarding clinical evidence), testing for characteristics considered important for probiotic efficacy could be indicative of possible mechanisms that underlie the observed clinical findings. Such characterizations could include survival at relevant body sites, the production of lactic acid or other short chain fatty acids, adhesion to mucus or intestinal epithelial cells, interaction with human immune cells, resistance to digestive enzymes, bile or acid, antibacterial activity via competitive exclusion or production of bacteriocins or hydrogen peroxide. To this end, the Belgian Superior Health Council published a useful report on approaches to characterize probiotics (Huys et al., 2013), focusing on identification, strain typing and safety assessment. However, it must be emphasized that such phenotypic characterization is not a requirement for probiotic status. These assays may provide indications of function or be useful in initial screening strategies, but they are not validated biomarkers of probiotic functionality. Strains possessing any or all of these characteristics cannot use the term probiotic solely based on the presence of these genotypes or phenotypes.

Recent meta-analyses have confirmed that available evidence for some health effects is most rigorously linked to specific strains of probiotics (McFarland and Evans, 2018; van den Akker et al., 2018). In some instances, health benefits may not be limited to specific strains, but can be shared among wider taxonomic groups (Sanders et al., 2017). This function may be linked to a single property of the probiotic microbe. An example is the presence of the enzyme lactase in the case of mediation of lactose intolerance symptoms. The presence of a trait such as this may be species rather than strain-specific. Even in the case of a health benefit mediated by a shared trait, the probiotic must be identified to the strain level.

## Probiotic Is Safe for Intended Use

Providing consumers with foods and dietary supplements that meet applicable safety standards is a basic responsibility of probiotic manufacturers. Establishing that a specific probiotic is safe for use in foods and dietary supplements requires, as a starting point, proper identification to the strain level, and further documenting safe use through historical evidence or experimentation. Historical data of safe use can be an important factor in an overall assessment of safety for an intended use. In its absence, safety must be determined based on scientific principles, including the conduct of adequate phase 1 studies (Brodmann et al., 2017). Strain safety is assessed on a case-by-case basis and no specific requirements for sufficient evidence can be made, but a probiotic strain needs to comply with the safety requirements stipulated by the national/regional regulator as discussed below.

### Probiotic Species and Strains Must Be Safe for Human Consumption

For daily use of probiotics by the general healthy population, potential safety concerns arising from the administration of live micro-organisms must be addressed. Many species of lactic acid bacteria, bifidobacteria and yeasts, representing most of the commercially available probiotic strains, are judged to be safe for use in foods and supplements. This is because they belong to genera and species with a documented history of safe use, either as probiotics or as starter cultures (Bourdichon et al., 2018). Going beyond history of safe use, the European Food Safety Authority (EFSA) has maintained lists of species presumed to be safe for human consumption in foods under the “Qualified Presumption of Safety” (QPS) concept since 2007 (EFSA, 2007). The QPS approach is an evidence-based, thorough and regularly updated approach to communicate on the safety of specific species of micro-organisms. The list is a reasonable basis for establishing safety of food strains when belonging to a QPS species, provided that the strain-specific testing described below is also conducted. The list results from historical data, from regular monitoring of the body of knowledge and through extensive scientific literature reviews, applied to a wide array of micro-organisms traditionally found in the food chain (EFSA, 2020b). It should be noted that the scope of QPS is food consumption by the general, healthy population and does not specifically take into consideration potential risks for vulnerable populations (EFSA, 2005) or non-food uses of probiotics. In addition, the QPS list is not exhaustive, as it is based on submissions to EFSA for premarket approval in the EU market, and many microorganisms used in traditional fermented foods are not included in the list (Bourdichon et al., 2018). In Europe, if a strain does not belong to a QPS species, the Novel Food regulation (EU, 2015) may apply before it can be brought to market. Other jurisdictions have other procedures to assess safety of probiotics, such as the generally recognized as safe (GRAS) regulation in the United States.

### Species- and Strain-Specific Safety Criteria for Probiotics

Any safety evaluation is predicated on proper species identification, according to the principles outlined in section “Introduction.” In addition, identification of genus or species-specific risk factors, and testing at strain level is required. Most important among these is the absence of acquired antimicrobial resistance genes or known virulence factors. Within the EU, EFSA has issued several guidelines describing phenotypic cut-off susceptibility and resistance values for relevant antibiotics and methods for determining these (EFSA, 2018). The guidelines should also be used for the assessment of bacterial and antimycotic susceptibility for yeasts (EFSA, 2018). Standardized analytical methods are available for the phenotypic screening of candidate bacterial strains (ISO-IDF, 2010). If resistance above cut-off values is observed, further characterization is required. WGS of the strain will confirm the presence or absence of known genes involved in the observed resistance. In cases where putative resistance genes are detected, it is recommended to determine if transposable elements are in their genomic vicinity. If this is the case it cannot be excluded that the resistance gene is transferable, and commercialization of the strain is not recommended. Otherwise, the genome sequence can assist in identifying putative antimicrobial resistance genes by searching at least two databases. For microorganisms not well-represented in databases, a Hidden-Markov model database is recommended. Depending on their taxonomy and their intended use, the strain’s genome may need to be assessed for the presence of genes coding for known virulence factors such as toxins, invasion, and adhesion factors (EFSA, 2020a). In those cases where antibiotic resistance cut-off values are not known, it would be the responsibility of the producer to ensure that the proposed probiotic strain(s) do not contain transferrable antibiotic resistance genes, and that the resistance profile is consistent with other members of the same species. In some cases it may be necessary to generate new data on the susceptibility profiles of the considered taxon, including making sure that susceptibility testing methods are relevant and adapted to the physiology of the considered micro-organisms.

Other phenotypic properties may be assessed at strain level for safety, such as the ability to form biogenic amines and D-lactate. Both can be conveniently tested through analysis of the genome or through standardized phenotypic tests. In addition, hemolytic activity and bile salt hydrolase activity are sometimes assessed at strain level, but their relevance to safety remains to be determined (Huys et al., 2013).

### *In vivo* Safety Tests

In the case of most current probiotics belonging to QPS species and with a documented history of safe use in foods, the value of *in vivo* safety tests is unclear, especially given the European Union’s position stating that for ethical and efficiency reasons, unnecessary research should not be performed on animals (EU, 2010). Little or no effect from QPS species is to be expected in healthy animal models, such as mice or rats (Shokryazdan et al., 2016).

Human intervention studies on the other hand allow for proper documentation of safety and tolerance of probiotics through rigorous monitoring and reporting of adverse events. Biological and clinical parameters, including vital signs, can be monitored to collect valuable safety data. Unexpected deviations from baseline or standard values might indicate a possible safety concern. Documenting these safety endpoints must be performed during any type of clinical intervention, analyzed and reported according to accepted scientific standards for human studies. Sponsors, investigators, authors, and journal editors should facilitate the systematic reporting of safety and tolerance data in human clinical interventions for probiotics. It should be noted that any study, particularly studies of longer duration and involving large numbers of subjects, will surely observe adverse events. The key point is to determine if the adverse events are different between the intervention groups (i.e., probiotic and placebo) and/or are considered to be intervention-related. To date, only rare, mild, and transient probiotic-related adverse events have been reported in studies with healthy subjects (Goldenberg et al., 2017). Specific sensitive populations exist (e.g., the young, old, pregnant, and immune compromised population) and medical supervision of probiotic intervention and use is advised in such populations (Sanders et al., 2016).

With a significant number of strains from *Lactobacillus*, *Bifidobacterium*, and yeast species having a long history of safe use and having been the subject of thorough assessments and monitoring, it can be concluded that there are no major safety concerns for their use in foods and dietary supplements for the general population. Safety evaluations focus on the intended use, which here is food and dietary supplements; other uses may have different safety requirements e.g., depending on their delivery format or dose.

## Probiotic Is Supported by at Least One Human Clinical Trial According to Generally Accepted Scientific Standards

The ability to confer a health benefit to the host is a fundamental part of the definition of a probiotic since 2001 (FAO/WHO, 2002) and was reaffirmed in 2014 (Hill et al., 2014). By health benefit we here mean a positive effect on some measure of a person’s health from, in this case, the use of probiotics. This phrasing is non-proscriptive by design, to allow innovation in exploring any number of possible health endpoints. At least one human trial demonstrating a health benefit is required to qualify the candidate microbial strain(s) for probiotic status, preferably followed by confirmatory trial(s). Herein, we qualify this requirement by stating that the trial must be conducted according to generally accepted scientific standards. In rare circumstances, as recognized by certain authorities, the term “probiotic” may be appropriately used by strains of a species (or other taxonomic group), where several members of that species have been shown to confer a benefit driven by a shared mechanism (Hill et al., 2014; Sanders et al., 2017). For example, lactase activity expressed by strains of *Streptococcus thermophilus* or *L. delbrueckii* subsp. *bulgaricus*, which leads to reduced symptoms associated with lactose maldigestion, is a common property of these species. Strains of *S. thermophilus* and *L. bulgaricus* can be considered “probiotic” based on this benefit. Further, to correctly use the term “probiotic” to describe such strains, the organism must be identified at strain level and shown to express the relevant trait. A valid demonstration of a health benefit depends both on the quality and soundness of the trial itself (i.e., how well it was designed and conducted) and on the capacity of the scientific community to critically appraise published trial results (i.e., how well it was reported). Several tools exist to facilitate the design, reporting, risk of bias (RoB) assessment and critical appraisal of clinical trials used to support probiotic status (Table 1).

**TABLE 1:** Tools to facilitate design, reporting, managing risk of bias, and critical appraisal of human intervention studies with probiotics.

Tool name	References and links
**Clinical trial protocol design guidelines**	
International council for harmonization of technical requirements for pharmaceuticals for human use E6 (R2)	https://database.ich.org/sites/default/files/E6_R2_Addendum.pdf
Statistical principles for clinical trials E9	https://database.ich.org/sites/default/files/E9_Guideline.pdf
Structure and content of clinical study reports E3	https://database.ich.org/sites/default/files/E3_Guideline.pdf
SPIRIT 2013	https://www.spirit-statement.org/
	https://www.spirit-statement.org/publications-downloads/
**Critical appraisal tools (quality assessment)**	
JBI checklists (Joanna Briggs Institute, 2017)	(Tufanaru et al., 2017) https://joannabriggs.org/ebp/critical_appraisal_tools, https://joannabriggs.org/sites/default/files/2019-05/JBI_RCTs_Appraisal_tool2017_0.pdf
CASP checklists (Critical appraisal skills program, 2018)	https://casp-uk.net/wp-content/uploads/2018/01/CASP-Randomised-Controlled-Trial-Checklist-2018.pdf
Critical appraisal checklists (SURE, 2018)	https://www.cardiff.ac.uk/__data/assets/pdf_file/0005/1142969/SURE-CA-form-for-RCTs-and-other-experimental-studies_2018.pdf
BMJ Best practice toolkit (BMJ publishing group limited, 2019)	https://bestpractice.bmj.com/info/us/toolkit/learn-ebm/appraising-2-armed-randomized-controlled-trials/
SIGN checklists and notes (Scottish Intercollegiate Guidelines Network, 2001-2019)	(Harbour and Miller, 2001) https://www.sign.ac.uk/assets/checklist_for_controlled_trials.doc
Critical appraisal tools (Centre for Evidence-Based Medicine, 2020)	https://www.cebm.net/wp-content/uploads/2018/11/RCT.pdf
EQUATOR (Enhancing the quality and transparency of health research) Network	https://www.equator-network.org
**Risk of Bias assessment tool**	
RoB 2 tool	(Sterne et al., 2019) https://methods.cochrane.org/bias/resources/rob-2-revised-cochrane-risk-bias-tool-randomized-trials, https://sites.google.com/site/riskofbiastool/welcome/rob-2-0-tool
**Reporting guidelines**	
CONSORT 2010	(Schulz et al., 2010) http://www.consort-statement.org/consort-2010

### Considerations for Protocol Design

Recognized guidelines for clinical trial design (and conduct) were originally developed to ensure participants’ welfare and ethical trial conduct and have been available and often mandatory for several decades, e.g., the Good Clinical Practice guidelines of the International Council for Harmonisation (ICH-GCP) and country-specific, legally binding versions (Vijayananthan and Nawawi, 2008). Compliance to ICH-GCP guidelines, in addition to the unequivocal ethical value it provides, also contributes to ensuring the generation of higher quality and more reliable data. An internationally recognized tool, endorsed by journals, funders, regulators and academic institutions worldwide, was developed specifically for the design of trial protocols that comply with the recommendations of the ICH-GCP, the WHO, and the International Committee of Medical Journal Editors (ICMJE). The “Standard Protocol Items: Recommendations for Interventional Trials (SPIRIT)” 2013 checklist contains a list of 33 elements that should be included in all clinical trial protocols (Chan et al., 2013). In accordance with the SPIRIT 2013 checklist (Item 2), the publication of the study protocol in a public database (e.g., ClinicalTrials.gov) prior to the start of the study is highly recommended^3^ and is viewed as a way of fostering the design of higher-quality studies while contributing to more transparent reporting of results. Furthermore, the publication of clinical trial protocols in peer-reviewed journals, which usually require that the protocol should be registered in a public registry, also constitutes a good practice that should be further encouraged in the probiotics field.

From a scientific standpoint, several trial design challenges frequently appear to prevent drawing formal conclusions about a health benefit in probiotics trials (Brussow, 2019). These may include details of the design of the study [randomized controlled trials (RCTs) vs. non-randomized trials; cross-over vs. parallel arms design], the participant’s allocation concealment, blinding (double-blind vs. single-blind or open label), the choice of controls (placebo vs. comparator treatment), the dosing and administration regimen (concentration used, administration schedule, start, and duration of the supplementation period), power calculations for the primary outcome, or the choice of population (health status, age, and gender). Due the inherent specificity of candidate microbial strains, no guideline specific for the whole probiotics field can be developed regarding the preference for a certain study design type, or of a specific dosing regimen over another. However, a careful consideration of these parameters in parallel with the microbial strain and target population characteristics is warranted at the study design stage (Shane et al., 2010). To this end, it may be helpful to gain prior knowledge of the accepted standards of trial reporting as well as of the tools available for the critical appraisal of published trials (Table 1).

### Considerations for Trial Reporting

Several international journals require authors to report the results of their trials according to an established and recognized set of guidelines, namely The Consolidated Standards of Reporting Trials (CONSORT), which has become the mainstay for reporting and publishing trial results (Schulz et al., 2010). While the 25-point CONSORT checklist was not created as a guideline for trial design and conduct, prior knowledge of the elements that must be reported can facilitate the design. The CONSORT 2010 checklist was considered during the development of the SPIRIT 2013 guideline for protocol design to facilitate the passage from SPIRIT-compliant protocol to a CONSORT-compliant report (Chan et al., 2013).

Compliance with the CONSORT 2010 guidelines for reporting trials will facilitate subsequent critical appraisal of the results and contribute to generating stronger conclusions from meta-analyses and systematic reviews. Briefly, CONSORT covers all aspects of trial design and conduct, data collection and analyses, as well as reporting. For example, CONSORT requires a participant flowchart explicitly stating numbers of participants for each step from recruitment to study completion, the exclusions, losses to follow up, and sizes of the intent-to-treat or per protocol populations. This information is crucial for future quality and RoB assessments needed afterward (e.g., systematic reviews for evidence-based medicine guidelines or regulatory purposes). CONSORT also stipulates that the authors should highlight the limitations of their study, such as the sources of bias and uncertainties that may influence the interpretation of the results. A description of results generalizability is expected, as well as a clear perspective of health benefits versus risks (implying a detailed reporting of the adverse events).

### Critical Appraisal and RoB Assessment of Published Trials

Critical appraisal and RoB assessment of clinical studies are important components of evidence-based medicine. They allow the determination, in an objective manner, of the weight of a trial’s findings (Buccheri and Sharifi, 2017). Numerous tools have been developed for these purposes, which are mostly designed for authors of systematic reviews and meta-analyses or best practice guidelines but can be useful when assessing the quality of trials that have been published without the use of a reporting guideline such as CONSORT 2010. This concerns trials published before 2010, but unfortunately also a number of more recent trials. Compliance to the CONSORT 2010 guidelines remains low in the medical literature in general (Jin et al., 2018).

The difference between critical appraisal (i.e., quality assessment) tools and RoB assessment tools may be considered as ambiguous, but the two approaches are clearly distinct. For example, the RoB assessment tools used by authors of Cochrane reviews are designed to address whether the results of the trial are free of bias and credible (Higgins et al., 2019; Sterne et al., 2019). On the other hand, quality assessment tools often include parameters relating both to reporting quality (e.g., obtaining ethical approval or describing power calculations) as well as to the quality, transparency, and consistency of the research (e.g., randomization and allocation concealment, proper control selection, and missing outcome data). The latter parameters are directly related to potential sources of bias assessed by RoB tools. Generally, the weight attributed to the results of a trial is proportional to how efficiently sources of bias have been avoided (Higgins et al., 2019). Considering that lack of randomization, blinding or controls are identified as significant sources of bias in clinical trials, double-blind RCTs have become the “gold standard” design to demonstrate health benefits in a reliable manner, as demonstrated by the higher score attributed to RCTs over other designs when grading the quality of evidence from clinical trials (Guyatt et al., 2008). Other study designs, such as open-label and uncontrolled studies, are possible and have been used in the past. While the results from such studies may not be robust enough to be used alone to qualify a probiotic designation, they can provide useful supportive documentation.

## Probiotic Is Alive in the Product at an Efficacious Dose Throughout Shelf Life

While the three previously described criteria refer specifically to a microbial strain to be considered as a probiotic, this fourth criterion applies to the product that delivers the probiotics. The definition of probiotics does not include a reference to a specific dose, but rather states that probiotics should be administered in amounts that are adequate to result in a health benefit for the host. Thus, it is conceivable, given that probiotics are living microbes capable of self-replication within the host, that over time a few probiotic cells could be sufficient to elicit a beneficial effect if they grow sufficiently within the host. This is certainly true for pathogenic microbes causing disease, which can cause deleterious effects on host health at extremely low doses because of their virulence and capacity to replicate within the host.

Dose ranging studies were intended to determine the tolerability, efficacy, and safety profile of an active substance that can be delivered in fixed concentrations and that normally cannot multiply post-administration (Ting, 2006). Consequently, dose ranging studies are a common feature in clinical trials but are less common in food and dietary supplement trials. This is largely a result of the presumption of safety for food ingredients. In clinical settings, dose ranging studies are usually performed after the maximum tolerable dose (MTD) has been elucidated for the bioactive under investigation. We are not aware of any oral MTD study that has been performed in humans for any probiotic strain or strain combination (section “Probiotic is Sufficiently Characterized”). Given that probiotics have an excellent safety profile and the fact that they have rarely been subjected to either MTD or dose ranging studies, it is common for most studies to simply choose a daily dose between 10^8^ and 10^11^ colony forming units (CFU), which reflect effective doses in past studies. While there may be an interest in determining an optimal dose that leads to a specific health benefit, this is not an essential criterion.

Quantification of the viability of probiotic strains should be done using standardized enumeration methods such as plating; CFU counting on selective growth media, e.g., for *Lactobacillus acidophilus* (ISO 20128; Table 2) and *Bifidobacterium* (ISO 29981 or IDF 220:2010; Table 2) or by flow cytometry (ISO 19344:2015; Table 2). Methods have been reviewed by various authors (Davis, 2014; Zielińska et al., 2018). The viability of probiotic strains at the efficacious dose should be documented in the test products during clinical investigations and guaranteed until the end of shelf life in commercial products according to quality procedures.

**TABLE 2:** Examples of standard methods for probiotic enumeration.

Standard	Taxon name	Web pages
ISO 20128:2006	*Lactobacillus acidophilus*	https://www.iso.org/obp/ui/#i so:std:iso:20128:ed-1:v1:en
ISO 29981 = IDF 220:2010	*Bifidobacterium*	https://www.iso.org/standard/45765.html
ISO 19344:2015 = IDF 232	Milk and milk products - Starter cultures, probiotics and fermented products - Quantification of lactic acid bacteria by flow cytometry	https://www.iso.org/obp/ui/#i so:std:iso:19344:ed-1:v1:en https://www.iso.org/standard/64658.html

For the quantification of probiotic combinations, culture-independent metagenomics methods based on high-throughput next-generation sequencing have been developed (Patro et al., 2016) although these methods do not ensure that what is counted is viable. These methods can reveal interesting information on potential contaminants (Quigley et al., 2013) but may lack methodological validation (Sohier et al., 2014). They can, however, be performed by accredited laboratories which guarantees a certain level of consistency and reproducibility. Molecular methods to determine viability in complex mixtures are under development such as e.g., propidium monoazide (PMA)-PCR (Scariot et al., 2018). These are, however, experimental and not standardized.

In general, fecal recovery is often used as a surrogate marker to reflect sufficient dose for gastrointestinal health targets. Dose ranging could be possible for those probiotics which have a readily determined endpoint of efficacy (e.g., serum cholesterol levels), although once again the issue of replication *in situ* could cause problems with interpretation. One study in which dose ranging was performed was an IBS trial involving *Bifidobacterium longum* subsp. *infantis* 35624 in which three doses were tested, 10^6^, 10^8^, and 10^10^ CFU. This had an interesting outcome in that the 10^8^ CFU dose was effective, whereas the other two doses were not. This anomalous result was likely because the capsules containing the highest dose did not dissolve, and therefore only the results of the other two doses could be assessed (Whorwell et al., 2006).

An alternative to classic dose ranging studies is to examine the large body of documented probiotic trials in humans and calculate the doses used in each study and the clinical outcomes. Such an analysis was recently performed, and the conclusions were nuanced (Ouwehand, 2017). For some probiotic/health state combinations there was evidence of a clear dose response, but for other combinations the data were not compelling.

If a particular study elicits the desired health benefit, then that dose would serve as the minimum dose for which a health claim should be permitted. Products using a higher dose should be also able to make the same claim, but claims should not be permitted for any dose lower than that tested in humans. Here, we will not further discuss health claims; that belongs in the realm of regulators. While regulators in general adhere to the probiotic definition, they tend to interpret the requirements differently in their respective jurisdictions. An analysis of this falls outside the scope of the current paper.

What influence, if any, the delivery format has on a probiotic effect is an interesting topic. To date, few studies have been conducted on the direct comparison of a probiotic delivered in different matrices related to the same clinical endpoint. Two reviews have addressed the matter with one concluding that there is currently no evidence that the delivery matrix has a substantial effect on probiotic efficacy (Sanders et al., 2014) and the other concluded that there may be strain dependent matrix effects (Flach et al., 2018). Both papers agree that data on the topic is scarce.

## Conclusion

Probiotics are the subject of global investigative research, innovative product design, effective marketing, regulatory scrutiny, focused consumer interest and use by healthcare practitioners. It would be beneficial to all involved in these undertakings to clearly understand the criteria needed for the word “probiotic” to be used responsibly. This paper describes the minimum criteria that apply to a probiotic strain that will be used in foods and dietary supplements and similar criteria may be applicable to other uses of probiotics. Specifically, the strain must be identified using recognized scientific methods, named according to valid current nomenclature, and named with a retrievable strain designation. Methods will vary depending on species of the probiotic and are likely to change as technologies evolve. Also, we recommend that it should be deposited in an international culture collection. Further, the strain must have demonstrated safety for its intended use and a demonstrated health benefit based on at least one study that meets generally accepted scientific standards or as per recommendations and provisions of local/national authorities when applicable. Sufficient levels of the probiotic strain(s) must be contained in the final product throughout the shelf life in order to be able to deliver the claimed (and evidence-based) health benefit. Products should be manufactured according to applicable good manufacturing requirements to assure safety, purity, and stability (Jackson et al., 2019) and should be labeled in a manner that communicates essential information on product contents (specific strains, level of live probiotic delivered at end of shelf life, and statements about health benefits as allowed) to the end-user. Adherence to these principles will assure that the marketplace does not contain products that misuse the term “probiotic.” Some local regulatory contexts can define probiotics in a different manner, but it is the responsibility of the product manufacturer to produce and market probiotics that follow local rules and regulations and are in line with the above defined principles.

## Author Contributions

All authors contributed to the conceptualization, wrote sections for the manuscript, reviewed and edited the manuscript, and read and agreed to the final version of the manuscript.

## Conflict of Interest

At the time of writing, SB and DO were employed by Danone Nutricia Research. EJ was employed by Chr. Hansen A/S. BP was employed by Yakult Europe BV. AT was employed by Rosell Institute for Microbiome and Probiotics. AO was employed by DuPont Nutrition and Biosciences. CH conducts academic research funded by commercial companies. MS consults with companies that manufacture probiotics or probiotic-containing products and serves as the executive science officer for ISAPP.

## Footnotes

1 https://www.bergeys.org/

2 http://www.ncbi.nlm.nih.gov/genbank/

3 http://www.icmje.org/news-and-editorials/clin_trial_sep2004.pdf
